# Effect of Extracurricular After-School Physical Activities on Academic Performance of Schoolchildren: A Cluster Randomized Clinical Trial

**DOI:** 10.1001/jamapediatrics.2023.3615

**Published:** 2023-09-18

**Authors:** Decai Wang, Ruilin Xiong, Jiaqing Zhang, Xiaotong Han, Ling Jin, Weijia Liu, Yabin Qu, Qianyun Chen, Shida Chen, Xiang Chen, Yuting Li, Mingguang He, Yangfa Zeng, Yizhi Liu

**Affiliations:** 1 State Key Laboratory of Ophthalmology, Zhongshan Ophthalmic Center, Sun Yat-sen University, Guangdong Provincial Key Laboratory of Ophthalmology and Visual Science, Guangdong Provincial Clinical Research Center for Ocular Diseases, Guangzhou, Guangdong, China; 2 School Health Unit, Guangzhou Center for Disease Control and Prevention, Guangzhou, China; 3 Guangdong Provincial Center for Disease Control and Prevention, Guangzhou, China; 4 Experimental Ophthalmology, The Hong Kong Polytechnic University, Hong Kong, China

## Abstract

**Importance:**

The beneficial effects of increasing outdoor physical activity time on children’s myopia onset and physical well-being are widely acknowledged. However, in countries with competitive educational systems, such as China, parents and school administrators may be relatively reluctant to increase the extracurricular physical activity time for children due to concerns that this action will compromise children’s academic performance.

**Objective:**

To investigate whether additional extracurricular physical activity time after school compromises the academic performance of schoolchildren.

**Design, Setting, and Participants:**

This cluster randomized clinical trial was conducted from October 2020 to June 2021 in Yudu, Jiangxi, China. Eligible children in grades 3 and 4 from 24 elementary schools were randomized to the intervention or control group. Primary analysis was conducted in the full sample using the intention-to-treat principle.

**Interventions:**

The intervention group received 2 hours of after-school physical activity time outdoors on school days. The control group was free to arrange their after-school activity.

**Main Outcomes and Measures:**

The primary outcome was the between-group mean difference in mathematics test scores at the end of 1 academic year, with a noninferiority margin of −3.3 points. Standardized mathematics tests, physical fitness assessments (in reference to the 2018 National Physical Fitness Survey Monitoring Programme in China), and cycloplegic autorefraction were performed at baseline and the end of 1 academic year. Myopia was defined as a cycloplegic spherical equivalent refraction of −0.5 diopters or less in either eye.

**Results:**

A total of 2032 children (mean [SD] age, 9.22 [0.62] years; 1040 girls [51.2%]) from 24 schools were randomized to the intervention group (12 schools; 1012 children) or control group (12 schools; 1020 children). The mean (SD) mathematics score at the end of 1 academic year was 78.01 (17.56) points in the intervention group and 77.70 (17.29) points in the control group. The adjusted between-group mean difference was 0.65 points (95% CI, −2.85 to 4.15). The adjusted between-group mean difference in physical fitness score was 4.95 points (95% CI, 3.56-6.34; *P* < .001) and −1.90% (95% CI, −18.72% to 14.91%; *P* > .99) in myopia incidence.

**Conclusions and Relevance:**

Results of this trial indicate that, compared with the control practice of free play after school, adding 2 hours of extracurricular physical activity outdoors after school was noninferior in academic performance and had superior efficacy in improving physical fitness.

**Trial Registration:**

ClinicalTrials.gov Identifier: NCT04587765

## Key Points

**Question:**

Will implementing a 2-hour extracurricular physical activity program after school compared with free use of after-school time compromise academic performance among schoolchildren in China?

**Findings:**

In this cluster randomized clinical trial of 24 elementary schools with 2032 participating children, adding 2 hours of extracurricular physical activity outdoors after school (intervention) did not result in significant differences in mathematics scores after 1 academic year compared with free use of after-school time (control) among children in grades 3 and 4.

**Meaning:**

Findings of this trial indicated that adding extracurricular physical activity outdoors was noninferior to free play after school and did not compromise children’s academic performance.

## Teaser

This cluster randomized clinical trial examines the difference in mathematics score, physical fitness, and myopia incidence between Chinese schoolchildren with free play activities after school and those with outdoor physical activities after school.

## Introduction

The beneficial effect of increased time outdoors on myopia control is widely acknowledged, and related school-based interventions have been implemented at a systemwide scale in many countries with varying levels of success.^1,2^ Additional benefits of more time outdoors include better physical, social, and emotional well-being.^3^ Given the global decline in physical fitness among children coupled with an increasing prevalence of childhood obesity,^4,5^ promoting more outdoor physical activities seems only advantageous.

However, in real-world scenarios, children are spending more time indoors in front of screens.^6,7^ Efforts to promote more physical activity outdoors are thwarted to some degree by parents and school administrators, especially in countries with highly competitive educational systems, such as China. Their major concern is that increased outdoor physical activity time will reduce studying time and hence compromise academic performance.^8,9^ To date, high-quality evidence regarding the effect of increasing outdoor physical activity at school on children’s academic performance remains inconclusive,^10,11^ and none of these studies originated from China.

To investigate whether additional extracurricular physical activity time after school compromises the academic performance of schoolchildren, we conducted this noninferiority cluster randomized clinical trial (RCT), with academic performance as the primary outcome. We hypothesized that, compared with the current practice in China of free play after school, adding extracurricular physical activity after school would not compromise children’s academic performance.

## Methods

### Study Design, Setting, and Participants

This unblinded, noninferiority, cluster RCT was conducted in Yudu County, Jiangxi Province, China. Baseline examinations were completed in October 2020, and a follow-up visit at the end of 1 academic year was completed in June 2021. All examinations were performed by the same study examiners using the same protocol and equipment. All study examiners (including D.W., R.X., J.Z., X.H., and S.C.) were qualified optometrists, ophthalmologists, or teachers. The trial protocol is provided in Supplement 1. The Ethics Committee of Zhongshan Ophthalmic Center at Sun Yat-sen University approved this study. This trial was conducted in accordance with the tenets of the Declaration of Helsinki.^12^ Written informed consent was obtained from children and their parents or guardians before participation. We followed the Consolidated Standards of Reporting Trials (CONSORT) reporting guideline.

A total of 24 elementary schools (12 rural and 12 urban) with a mean class size of more than 30 children and sufficient conditions for 2 or more classes to play sports at the same time were invited and agreed to participate. One class each in grades 3 and 4 was randomly selected. Children were between ages 7 and 11 years. Children were excluded if they were unable to participate on 2 or more school days or were unsuitable for physical activity due to health or physical restrictions.

### Randomization, Blinding, and Intervention

School-based cluster randomization was conducted by an independent data analyst (L.J.) using a random number-generating program. All 24 schools were stratified into 6 strata based on location (rural or urban) and mathematics scores (high, medium, or low). Schools in each stratum were randomized to the intervention or control group (Figure). Due to the nature of this intervention, blinding was unfeasible. However, all study examiners (including D.W., R.X., J.Z., X.H., and S.C.) and the statistician (L.J.) were blinded to study group assignment.

**Figure.**
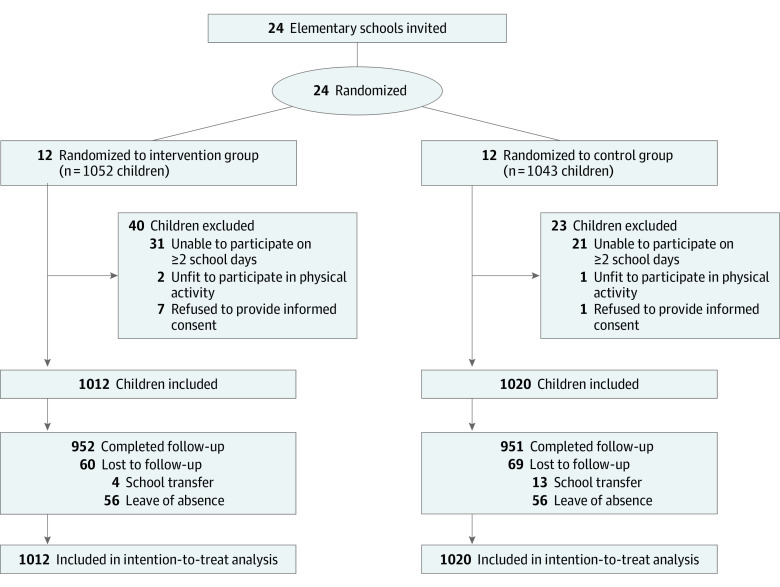
Recruitment and Flow of Participants

Curriculums were scheduled in 2 separate semesters with approximately a 1-month break in between, during which the study intervention was suspended. Children in the control group were free to arrange their after-school time after 4 pm. Children in the intervention group received an additional 2-hour (4-6 pm) extracurricular physical activity program after school on school days, guided and monitored by their head teachers and physical education teachers. Before study commencement, the Education Bureau of Yudu helped consolidate the school sports facilities, and a professional team organized by Zhongshan Ophthalmic Center and the local education bureau delivered a 1-day intensive training session for teachers, detailing the specific requirements and providing guidance. Recommended physical activity programs included but were not limited to basketball, soccer, badminton, and table tennis. In cases of bad weather when outdoor physical activity was unfeasible, physical activity occurred in semiopen areas or indoors instead. The teachers could decide on the specific types of physical activity programs as appropriate and were asked to fill out diary forms to record the daily implementation status.

### Outcomes

All outcomes were measured at baseline and the end of 1 academic year. The primary outcome was the between-group mean difference in mathematics test scores after 1 academic year. Mathematics tests were administered at school on the same day. Unified test papers were designed with professional assistance from the Education Bureau of Yudu in accordance with the design principles of the International Mathematics and Science Study^13^ and the local unified examination papers. The tests were scored by trained teachers according to unified answers and scoring procedures. The original scores ranged from 0 to 100 points and were further divided into 5 levels: A+ (≥95 to ≤100 points), A (≥85 to <95 points), B (≥75 to <85 points), C (≥60 to <75 points), and D (<60 points). We double-checked the mathematics scores and levels.

Secondary outcomes were overall physical fitness score and myopia incidence after 1 academic year. Physical fitness scores were assessed at school by trained examiners from Zhongshan Ophthalmic Center, with reference to the 2018 National Physical Fitness Survey Monitoring Programme in China. Height and weight were measured using a height and weight scale and with children standing in light clothing and without shoes. Body mass index was calculated as weight in kilograms divided by height in meters squared. Lung vital capacity was measured by a spirometer. Fifty-meter sprint, sit-and-reach test, 1-minute sit-up, and 1-minute jump rope were conducted. The overall physical fitness score was calculated as the sum of weighted individual scores, with age- and sex-stratified weights of body mass index at 15%, lung vital capacity at 15%, 50-m sprint at 20%, sit-and-reach test at 20%, 1-minute sit-up at 10%, and 1-minute jump rope at 20%. These weights were derived from the Chinese National Student Physical Fitness Standard, a norm testing battery in China.^14,15^ Physical fitness scores ranged from 0 to 100 points, with higher scores indicating better fitness.

Ocular examinations were performed at school by ophthalmologists and optometrists from Zhongshan Ophthalmic Center. Axial length was measured using an optical biometer (IOLMaster; Carl Zeiss Meditec), and measurements were repeated 5 times for each eye. Cycloplegia was achieved with 3 drops of 1% cyclopentolate administered at 0, 5, and 20 minutes. Cycloplegia was considered to be complete if the pupillary light reflex was absent and the pupil was dilated to at least 6 mm. Cycloplegic autorefraction (KR 8800; Topcon) was performed and repeated 3 times for each eye. Spherical equivalent refraction (SER) was calculated as the spherical power plus half cylindrical power. Myopia was defined as a cycloplegic SER of −0.5 D (diopter) or less in either eye.^16^ Incidence of myopia was defined as the proportion of children with myopia after 1 academic year among those without myopia at baseline.

### Questionnaire 

A behavior-related questionnaire derived from a previous RCT^1^ was completed by children under the supervision of their parents or guardians at baseline and the end of 1 academic year. Information on wake-up time and bedtime at night, nap time, outdoor time, study or reading time, and screen time was collected. Study or reading time was defined as the sum of after-school time spent on additional lessons, homework, and reading. Screen time was considered as the sum of time spent watching television or using computers, game consoles, and cellular or smartphones. The 5-item World Health Organization Well-Being Index was used to assess the psychological well-being of children,^17^ with an overall score range from 0 (no well-being) to 25 (maximum well-being).

### Sample Size

The sample size was calculated based on the primary outcome and a noninferiority margin of −3.3 points. Based on the historical data obtained from the schools before the trial, the median academic score distribution was level B (≥75 to <85 points). Therefore, a decrease of 10 points was expected to change the final mathematics performance from level B to C. The noninferiority margin (the level at which the intervention was noninferior to the control) was chosen as one-third of the range of 10 points. The sample size estimation was conducted based on the assumption of a 1-sided α = .025, 90% power, an intraclass correlation of 0.10, an SD of 7 for mathematics scores, a mean cluster size of 60 children (2 classes per cluster with a class size of 30), and less than 5% of children being lost to follow-up. The sample size required was 12 schools per group or a total sample size of 24 schools. The PASS software, version 16.0 (NCSS) was used for the calculation.

### Statistical Analyses

The main analysis was conducted in the full sample using the intention-to-treat principle except for the myopia incidence analysis, from which children with cycloplegia failure and ocular abnormalities were excluded. Missing values were imputed by multiple imputation.^18^ This approach created 20 copies of the data, in which missing values were imputed by chained equations. Results were obtained by averaging these 20 data sets using Rubin rules.

Continuous and categorical data were reported as means (SDs) and numbers (proportions), respectively. The normality in the distribution of continuous data was checked using the Kolmogorov-Smirnov normality test and histogram. All analyses took into account the cluster effects within schools. Baseline distributions of continuous (age, height, weight, and sleep time), binary (sex), and ordered categorical (outdoor time, screen time, and study or reading time) variables between the intervention and control groups were compared using linear, logistic, and ordinal logistic regression models, respectively. The statistical inference was determined on whether the lower limit of the 95% CI of the between-group mean difference in the primary outcome was no less than the predefined noninferiority margin. Linear regression models assessed the differences between the 2 study groups in mathematics scores, physical fitness scores, SER, and axial length at baseline and the end of 1 academic year and the changes in these variables after 1 academic year. Differences in the prevalence and incidence of myopia were compared by proportional *Z* tests.

Sensitivity analyses were performed using the per-protocol principle, which included only children who completed the intervention as originally assigned without any major protocol violation and children who completed the follow-up visit. Bonferroni correction was applied to multiple comparisons for secondary outcomes (physical fitness score and myopia incidence), and corrected *P* values were presented. Two-sided *P* < .05 indicated statistical significance. All analyses were performed using Stata 16 (StataCorp LLC).

## Results

A total of 24 elementary schools with 2095 children were screened for eligibility. After excluding 63 children who were unable to participate on 2 or more school days, were unsuitable for physical activity, or refused to provide informed consent, 2032 children (1040 girls [51.2%] and 992 boys (48.8%) with a mean (SD) age of 9.22 [0.62] years) were included and randomized to the intervention group (12 schools with 1012 children) or the control group (12 schools with 1020 children). Of these children, 1903 (93.7%) completed the follow-up visit and 129 (6.3%) were lost to follow-up due to school transfer or leave of absence (Figure). Table 1 illustrates the baseline characteristics of children in the intervention and control groups, which were well balanced.

**Table 1.** Baseline Characteristics of Children in the Intervention and Control Groups^a^

Characteristic	Children, No. (%)	
	Intervention group	Control group
All children	1012 (49.8)	1020 (50.2)
**Demographics**		
Age, mean (SD), y	9.21 (0.62)	9.23 (0.62)
Sex		
Female	501 (49.5)	539 (52.8)
Male	511 (50.5)	481 (47.2)
Height, mean (SD), cm	131.45 (6.74)	130.56 (7.03)
Weight, mean (SD), kg	28.13 (5.92)	28.49 (5.98)
**Behaviors after school^b^**		
Outdoor time, h/d		
≤0.5	472 (46.7)	448 (44.0)
>0.5 to ≤1.0	348 (34.4)	329 (32.3)
>1.0 to ≤1.5	140 (13.9)	136 (13.4)
>1.5	51 (5.0)	106 (10.4)
Screen time, h/d		
≤0.5	755 (74.7)	753 (73.9)
>0.5 to ≤1.0	175 (17.3)	166 (16.3)
>1.0 to ≤1.5	49 (4.9)	54 (5.3)
>1.5	32 (3.2)	46 (4.5)
Study or reading time, h/d		
≤1.0	703 (70.0)	773 (77.0)
>1.0 to ≤1.5	117 (11.6)	127 (12.6)
>1.5 to ≤2.0	112 (11.1)	58 (5.8)
>2.0	73 (7.3)	46 (4.6)
Sleep time, mean (SD), h/d	9.45 (0.94)	9.41 (0.97)

^a^ Distributions of continuous (age, height, weight, and sleep time), binary (sex), and ordered categorical (outdoor time, screen time, and study or reading time) variables between the intervention and control groups were compared using linear, logistic, and ordinal logistic regression models, respectively. All analyses took cluster effects within schools into account.

^b^ Missing values were outdoor time and screen time (n = 2), study or reading time (n = 23), and sleep time (n = 18).

Overall, 4548 (96.3%) of the 4724 scheduled extracurricular physical activity sessions were successfully implemented. Some sessions could not be implemented due to inclement weather (144 [3.0%]) or school events (32 [0.7%]). A total of 3626 sessions (79.7%) were implemented outdoors, whereas 518 sessions (11.4%) were implemented in semiopen spaces and 404 (8.9%) occurred indoors (eTables 1 and 2 in Supplement 2).

At baseline, mathematics scores (mean difference, −0.59; 95% CI, −5.13 to 3.95), overall physical fitness scores (mean difference, 0.73; 95% CI, −0.86 to 2.33), and myopia prevalence (mean difference, −0.57; 95% CI, −19.64 to 18.49) were similar in the 2 groups (Table 2 and Table 3). The mean (SD) mathematics score at the end of 1 academic year was 78.01 (17.56) points in the intervention group and 77.70 (17.29) points in the control group, with an adjusted between-group mean difference of 0.65 points (95% CI, −2.85 to 4.15) (Table 2). The lower limit of the 95% CI of the mean difference was greater than the margin of −3.3 points, indicating noninferiority.

**Table 2.** Intention-to-Treat Analysis of Changes in Mathematics Test and Physical Fitness Scores After 1 Academic Year Between the Intervention and Control Groups

Variable^a^	Study group, mean (SD)		Mean difference (95% CI)	*P* value^b^
	Intervention	Control		
All children	1012 (49.8)	1020 (50.2)		
Mathematics scores				
Baseline	73.81 (17.92)	74.40 (16.97)	−0.59 (−5.13 to 3.95)	
1 Academic year	78.01 (17.56)	77.70 (17.29)	0.65 (−2.85 to 4.15)	.71
Change from baseline	4.16 (17.01)	3.36 (14.48)	0.67 (−3.07 to 4.41)	
Physical fitness scores				
Overall				
Baseline	69.48 (8.97)	68.75 (9.02)	0.73 (−0.86 to 2.33)	
1 Academic year	81.49 (6.26)	76.24 (7.29)	4.95 (3.56 to 6.34)	<.001^c^
Change from baseline	11.95 (7.32)	7.59 (7.73)	4.67 (3.17 to 6.18)	
BMI				
Baseline	95.61 (9.39)	94.61 (10.75)	1.00 (−0.53 to 2.54)	NA
1 Academic year	95.35 (9.98)	95.38 (10.37)	−0.72 (−1.57 to 0.12)	
Change from baseline	−0.28 (7.89)	0.78 (8.19)	−0.75 (−1.60 to 0.10)	
Lung vital capacity				
Baseline	75.52 (11.34)	77.28 (10.75)	−1.76 (−5.17 to 1.65)	NA
1 Academic year	82.21 (8.86)	77.28 (12.16)	5.69 (3.93 to 7.44)	
Change from baseline	6.70 (10.70)	0.11 (12.24)	5.61 (3.75 to 7.47)	
50-m sprint				
Baseline	63.13 (18.24)	64.08 (16.87)	−0.94 (−5.47 to 3.58)	NA
1 Academic year	77.24 (14.17)	72.20 (15.59)	5.37 (2.17 to 8.57)	
Change from baseline	14.20 (17.89)	7.98 (17.93)	5.30 (1.70 to 8.91)	
Sit-and-reach test				
Baseline	71.61 (15.14)	71.10 (14.84)	0.51 (−1.91 to 2.92)	NA
1 Academic year	85.09 (9.81)	76.11 (10.53)	8.83 (6.07 to 11.59)	
Change from baseline	13.39 (14.71)	12.84 (5.10)	8.77 (5.82 to 11.72)	
1-min Jump rope				
Baseline	53.83 (25.42)	48.84 (26.58)	5.00 (−1.12 to 11.12)	NA
1 Academic year	75.90 (13.83)	68.93 (17.74)	5.81 (2.08 to 9.55)	
Change from baseline	21.81 (24.07)	20.14 (25.46)	5.69 (1.73 to 9.66)	
1-min sit-ups				
Baseline	60.99 (20.56)	61.62 (19.78)	−0.64 (−5.13 to 3.86)	NA
1 Academic year	72.14 (16.07)	68.83 (14.73)	3.56 (0.69 to 6.42)	
Change from baseline	11.02 (18.36)	7.20 (16.96)	3.56 (0.50 to 6.62)	

Abbreviations: BMI, body mass index (calculated as weight in kilograms divided by height in meters squared); NA, not applicable.

^a^ Primary outcome was the between-group mean difference in mathematics test scores after 1 academic year. Secondary outcomes were overall physical fitness scores and myopia incidence after 1 academic year. The remaining outcomes in this table were exploratory outcomes.

^b^ Normality in the distribution of continuous data was checked using the Kolmogorov-Smirnov normality test. Linear regression models assessed the differences between the 2 groups in the mathematics test scores and physical fitness scores at baseline and at the end of 1 academic year, as well as changes in these variables after 1 academic year. All analyses took into account the cluster effects within schools.

^c^ Bonferroni correction was applied to multiple comparisons for secondary outcomes (physical fitness score and myopia incidence), and the corrected *P* value was presented.

**Table 3.** Intention-to-Treat Analysis of Changes in Incident Myopia and Relevant Ocular Parameters After 1 Academic Year Between the Intervention and Control Groups^a^

Variable^b^	Study group, No. (%)		Mean difference (95% CI)	*P* value^c^
	Intervention	Control		
All children	798 (48.9)	833 (51.1)		NA
Prevalence of myopia				
Baseline	96 (12.0)	105 (12.6)	−0.57 (−19.64 to 18.49)	NA
1 Academic year	154 (19.3)	179 (21.5)	−2.19 (−35.50 to 30.93)	NA
Incidence of myopia	58/702 (8.3)	74/728 (10.2)	−1.90 (−18.72 to 14.91)	>.99^d^
SER, mean (SD), diopter				
Baseline	0.63 (1.05)	0.55 (1.13)	0.08 (−0.06 to 0.22)	NA
1 Academic year	0.35 (1.22)	0.23 (1.30)	0.04 (−0.06 to 0.13)	NA
Change from baseline	−0.28 (0.37)	−0.32 (0.47)	0.03 (−0.06 to 0.13)	NA
Axial length, mean (SD), mm				
Baseline	23.10 (0.77)	23.11 (0.75)	−0.001 (−0.10 to 0.09)	NA
1 Academic year	23.28 (0.82)	23.31 (0.80)	−0.03 (−0.06 to 0.01)	NA
Change from baseline	0.17 (0.14)	0.20 (0.14)	−0.03 (−0.07 to 0.01)	NA

Abbreviations: NA, not applicable; SER, spherical equivalent refraction.

^a^ Children with ocular abnormalities at baseline (n = 37) and follow-up visit (n = 42) were excluded from the analysis. Corresponding figures for unsuccessful cycloplegia were 197 and 125, respectively.

^b^ Myopia incidence after 1 academic year was a secondary outcome. The remaining outcomes in this table were exploratory outcomes.

^c^ The differences in the prevalence and incidence of myopia were compared by proportional *Z* tests. Linear regression models assessed the differences between the 2 study groups in the SER and axial length at baseline and at the end of 1 academic year, as well as changes of these variables after 1 academic year (adjusting for the baseline SER or axial length). All analyses took into account the cluster effects within schools.

^d^ Bonferroni correction was applied to multiple comparisons for secondary outcomes (physical fitness score and myopia incidence), and the corrected *P* value was presented.

The mean (SD) overall physical fitness score at the end of 1 academic year was 81.49 (6.26) points in the intervention group and 76.24 (7.29) points in the control group, with an adjusted mean difference of 4.95 points (95% CI, 3.56-6.34; *P* < .001) (Table 2). The physical fitness score improvement during the follow-up was 4.67 points (95% CI, 3.17-6.18) higher in the intervention group than in the control group.

After 1 academic year, children in the intervention group (58 of 702 [8.3%]) had less incident myopia than those in the control group (74 of 728 [10.2%). However, the adjusted mean difference did not reach statistical significance (−1.90%; 95% CI, −18.72% to 14.91%; *P* > .99) (Table 3).

During the follow-up, the proportion of children with screen time greater than 30 minutes per day increased by 1.7% (from 25.3% at baseline to 27.0% after 1 academic year) in the intervention group and 5.4% (from 26.1% at baseline to 31.5% after 1 academic year) in the control group. Time spent on studying or reading was not significantly different between the 2 groups (mean [SD], 55.65 [40.33] vs 49.17 [30.46] min/d). Specifically, there was no significant difference in mathematics scores between children who spent more than 2 hours studying or reading after school and children who spent less than 1 hour (β = −1.58; 95% CI, −4.46 to 1.31). The overall well-being index of children was similar at baseline and after 1 academic year in both the intervention group (mean [SD], 16.9 [5.11] vs 16.8 [4.44] points) and control group (mean [SD], 16.2 [5.87] vs 16.9 [6.00] points). Sensitivity analyses based on the per-protocol principle had results similar to those of the primary analyses (eTable 3 in Supplement 2).

## Discussion

In this cluster RCT, children who received an additional 2-hour extracurricular physical activity after school showed noninferior academic performance compared with those who were free to arrange their after-school time over 1 academic year. Children who received extracurricular physical activity had significantly higher physical fitness scores than the control group.

Existing RCTs regarding the effect of increasing physical activity on academic performance are limited and have substantial heterogeneity in sample size, participant characteristics, and study duration. A meta-analysis reported that physical activity could improve mathematics scores by 1.12 points (95% CI, 0.56-1.67) compared with controls, but 3 of the 4 included RCTs had a follow-up time shorter than 2 months.^19^ There is inconsistent evidence from other studies with longer follow-ups regarding the effect of adding physical activity time on academic performance, with 2 trials^20,21^ reporting no significant effect and 3 trials^22,23,24^ finding a beneficial effect on children’s mathematics performance in elementary schools.

The findings from this trial showed that participation in after-school extracurricular physical activity for 1 academic year did not compromise academic performance. Success in academic performance was reported to be associated with well-being and the cognitive functions that contribute to the processes of memory, perception, intellect, and action.^11,25,26,27^ Many psychosocial (eg, enhanced social connectedness and support for positive moods), neurobiological (eg, improved gray matter volume and activation and release of endogenous opioids), and behavioral (eg, improved sleep quality, self-regulation, and coping skills) mechanisms may play roles in enhancing cognitive and mental health following physical activity,^28^ a finding that warrants further investigation.

Consistent with previous research, 2-hour extracurricular physical activity after school was noted to be beneficial for physical fitness in this study. Data from the 2014 National Survey on Students’ Constitution and Health suggested that children who engaged in more than 1 hour of physical activity per day had a 0.41-fold higher passing rate on the composite physical fitness test than those with less than 1 hour of physical activity.^29^ A systematic review of 89 studies also indicated that school-based physical activity interventions were associated with improved physical fitness, which was reported as maximal oxygen uptake in children and adolescents.^30^

The effect of time outdoors on myopia prevention has been well-established in existing studies.^1,31,32^ However, outdoor physical activity did not significantly reduce incident myopia in this trial, which may be primarily attributed to the relatively short follow-up. This trial was initially designed to assess myopia incidence at the end of 2 academic years. However, the local government in Yudu learned of the beneficial impact of extracurricular physical activity after the project seminar and promptly issued a government policy guideline recommending the implementation of 2-hour extracurricular physical activity throughout the county. Due to this unforeseen development, it became impractical to maintain a control group as originally planned. This trial had to be terminated before the initially scheduled completion.

A common parental concern is that more physical activity time leads to reduced studying time. Results of this trial largely alleviate this concern by providing evidence that time spent on after-school studying or reading was not significantly different between the intervention and control groups. In addition, mathematics scores did not differ significantly between children with more than 2 hours vs children with less than 1 hour of studying or reading after school. The proportion of children with screen time of more than 30 minutes per day increased more in the control group than the intervention group, suggesting that more after-school outdoor physical activity may provide extra benefits in reducing screen time.

### Strengths and Limitations

To our knowledge, this study is the first RCT to assess the effect of a 2-hour extracurricular physical activity program on academic performance in Chinese children over 1 academic year. Other strengths of the trial included a large sample size, high retention rate, and refraction tests after full cycloplegia to evaluate myopia status.

This trial also has several limitations. First, it was difficult to unify the type and intensity of physical activity and equip all participating children with wearable devices for objective, real-time testing due to the unevenness of school facilities and the large sample size. Second, only mathematics test scores were used to evaluate the academic performance; further studies of the effect of physical activity on performance in other academic disciplines are needed. Third, only a questionnaire-based well-being index of children was assessed; more comprehensive mental health assessments after physical activity program intervention are needed in the future. Fourth, as this study focused on children in grades 3 and 4 in a single country, the generalizability of the findings to children in other countries with other ethnicities and age groups is limited, and caution should be taken when generalizing the results.

## Conclusions

The findings of this RCT suggested that adding 2 hours of extracurricular physical activity per day after school was noninferior to the current practice of free play after school in China regarding academic performance. This intervention had a beneficial effect on physical fitness of schoolchildren.

## Notes

Supplement 1. Trial Protocol and Statistical Analysis

Supplement 2. **eTable 1.** Compliance With the Extracurricular Physical Activities in the Intervention Group **eTable 2.** Percentages of Extracurricular Physical Activities Implemented Outdoors, Indoors, and in Semi-Open Spaces in the Intervention Group **eTable 3.** Per-Protocol Analysis of Changes in Mathematic Scores and Physical Fitness After 1 Academic Year Between the Intervention and Control Groups

Supplement 3.
Data Sharing Statement

## References

[1] He M, Xiang F, Zeng Y, . Effect of time spent outdoors at school on the development of myopia among children in China: a randomized clinical trial. JAMA. 2015;314(11):1142-1148. doi:10.1001/jama.2015.10803 26372583

[2] Morgan IG, Jan CL. China turns to school reform to control the myopia epidemic: a narrative review. Asia Pac J Ophthalmol (Phila). 2022;11(1):27-35. doi:10.1097/APO.0000000000000489 35044336

[3] Hills AP, Dengel DR, Lubans DR. Supporting public health priorities: recommendations for physical education and physical activity promotion in schools. Prog Cardiovasc Dis. 2015;57(4):368-374. doi:10.1016/j.pcad.2014.09.010 25269062

[4] Wang S, Dong YH, Wang ZH, Zou ZY, Ma J. Trends in overweight and obesity among Chinese children of 7-18 years old during 1985-2014. Article in Chinese. Zhonghua Yu Fang Yi Xue Za Zhi. 2017;51(4):300-305. doi:10.3760/cma.j.issn.0253-9624.2017.04.005 28395462

[5] Dong YH, Liu HB, Wang ZH, . Prevalence of myopia and increase trend in children and adolescents aged 7-18 years in Han ethnic group in China, 2005-2014. Article in Chinese. Zhonghua Liu Xing Bing Xue Za Zhi. 2017;38(5):583-587. doi:10.3760/cma.j.issn.0254-6450.2017.05.005 28651391

[6] Hedderson MM, Bekelman TA, Li M, ; Environmental Influences on Child Health Outcomes Program. Trends in screen time use among children during the COVID-19 pandemic, July 2019 through August 2021. JAMA Netw Open. 2023;6(2):e2256157. doi:10.1001/jamanetworkopen.2022.56157 36790805 PMC9932850

[7] Delisle Nyström C, Carlander A, Cassel S, Rosell M, J-Son Höök M, Löf M. Physical activity and screen time in Swedish children and adolescents: the generation pep study 2018-2021. Acta Paediatr. 2023;112(3):460-468. doi:10.1111/apa.16594 36371645 PMC10098717

[8] Patchen AK, Rakow DA, Wells NM, Hillson S, Meredith GR. Barriers to children’s outdoor time: teachers’ and pricipals’ experience in elementary schools. Environ Educ Res. Published online July 12, 2022. Accessed August 5, 2023 doi:10.1080/13504622.2022.2099530

[9] Mart M. Parental perceptions to outdoor activities. Int J Prog Educ. 2021;17(4):358-372. doi:10.29329/ijpe.2021.366.22

[10] Howie E, Pate R. Physical activity and academic achievement in children: a historical perspective. J Sport Health Sci. 2012;1:160-169. doi:10.1016/j.jshs.2012.09.003

[11] Donnelly JE, Hillman CH, Castelli D, . Physical activity, fitness, cognitive function, and academic achievement in children: a systematic review. Med Sci Sports Exerc. 2016;48(6):1197-1222. doi:10.1249/MSS.0000000000000901 27182986 PMC4874515

[12] World Medical Association. World Medical Association Declaration of Helsinki: ethical principles for medical research involving human subjects. JAMA. 2013;310(20):2191-2194. doi:10.1001/jama.2013.281053 24141714

[13] Martin MO. Third International Mathematics and Science Study: an overview. In: Martin MO, Kelly DL, eds. Third International Mathematics and Science Study (TIMSS) Technical Report, Volume I: Design and Development. Boston College; 1996.

[14] Ministry of Education of the People’s Republic of China Notice of the Ministry of Education on the National Student Physical Fitness Standard. 2014. Accessed August 5, 2023. http://www.moe.gov.cn/s78/A17/twys_left/moe_938/moe_792/s3273/201407/t20140708_171692.html

[15] Zhu Z, Yang Y, Kong Z, Zhang Y, Zhuang J. Prevalence of physical fitness in Chinese school-aged children: findings from the 2016 Physical Activity and Fitness in China-the Youth Study. J Sport Health Sci. 2017;6(4):395-403. doi:10.1016/j.jshs.2017.09.003 30356643 PMC6189247

[16] Wolffsohn JS, Kollbaum PS, Berntsen DA, . IMI: clinical myopia control trials and instrumentation report. Invest Ophthalmol Vis Sci. 2019;60(3):M132-M160. doi:10.1167/iovs.18-25955 30817830

[17] Topp CW, Østergaard SD, Søndergaard S, Bech P. The WHO-5 Well-Being Index: a systematic review of the literature. Psychother Psychosom. 2015;84(3):167-176. doi:10.1159/000376585 25831962

[18] Azur MJ, Stuart EA, Frangakis C, Leaf PJ. Multiple imputation by chained equations: what is it and how does it work? Int J Methods Psychiatr Res. 2011;20(1):40-49. doi:10.1002/mpr.329 21499542 PMC3074241

[19] Meli AM, Ali A, Mhd Jalil AM, Mohd Yusof H, Tan MMC. Effects of physical activity and micronutrients on cognitive performance in children aged 6 to 11 years: a systematic review and meta-analysis of randomized controlled trials. Medicina (Kaunas). 2021;58(1):57. doi:10.3390/medicina58010057 35056365 PMC8781636

[20] Sallis JF, McKenzie TL, Kolody B, Lewis M, Marshall S, Rosengard P. Effects of health-related physical education on academic achievement: Project SPARK. Res Q Exerc Sport. 1999;70(2):127-134. doi:10.1080/02701367.1999.10608030 10380244

[21] Coe DP, Pivarnik JM, Womack CJ, Reeves MJ, Malina RM. Effect of physical education and activity levels on academic achievement in children. Med Sci Sports Exerc. 2006;38(8):1515-1519. doi:10.1249/01.mss.0000227537.13175.1b 16888468

[22] Donnelly JE, Greene JL, Gibson CA, . Physical Activity Across the Curriculum (PAAC): a randomized controlled trial to promote physical activity and diminish overweight and obesity in elementary school children. Prev Med. 2009;49(4):336-341. doi:10.1016/j.ypmed.2009.07.022 19665037 PMC2766439

[23] Shephard RJ. Habitual physical activity and academic performance. Nutr Rev. 1996;54(4 pt 2):S32-S36. doi:10.1111/j.1753-4887.1996.tb03896.x 8700451

[24] Gao Z, Hannan P, Xiang P, Stodden DF, Valdez VE. Video game-based exercise, Latino children’s physical health, and academic achievement. Am J Prev Med. 2013;44(3) (suppl 3):S240-S246. doi:10.1016/j.amepre.2012.11.023 23415189

[25] Cárdenas D, Lattimore F, Steinberg D, Reynolds KJ. Youth well-being predicts later academic success. Sci Rep. 2022;12(1):2134. doi:10.1038/s41598-022-05780-0 35136114 PMC8826920

[26] Gräbel BF. The relationship between wellbeing and academic achievement: a systematic review. 2017. Accessed August 5, 2023. https://essay.utwente.nl/73514/

[27] Peng P, Kievit RA. The development of academic achievement and cognitive abilities: a bidirectional perspective. Child Dev Perspect. 2020;14(1):15-20. doi:10.1111/cdep.12352 35909387 PMC7613190

[28] Lubans D, Richards J, Hillman C, . Physical activity for cognitive and mental health in youth: a systematic review of mechanisms. Pediatrics. 2016;138(3):e20161642. doi:10.1542/peds.2016-1642 27542849

[29] Zhang JS, Yan XJ, Hu PJ, . Analysis on the trend of prevalence of excellent and good physical fitness and health status among Chinese Han students aged 13 to 18 years and related influencing factors from 1985 to 2014. Article in Chinese. Zhonghua Yu Fang Yi Xue Za Zhi. 2020;54(9):981-987. doi:10.3760/cma.j.cn112150-20191121-00877 32907289

[30] Neil-Sztramko SE, Caldwell H, Dobbins M. School-based physical activity programs for promoting physical activity and fitness in children and adolescents aged 6 to 18. Cochrane Database Syst Rev. 2021;9(9):CD007651. doi:10.1002/14651858.CD007651.pub3 34555181 PMC8459921

[31] Wu PC, Chen CT, Lin KK, . Myopia prevention and outdoor light intensity in a school-based cluster randomized trial. Ophthalmology. 2018;125(8):1239-1250. doi:10.1016/j.ophtha.2017.12.011 29371008

[32] Jin JX, Hua WJ, Jiang X, . Effect of outdoor activity on myopia onset and progression in school-aged children in northeast China: the Sujiatun Eye Care Study. BMC Ophthalmol. 2015;15:73. doi:10.1186/s12886-015-0052-9 26152123 PMC4495846

