# Supplementary material for: Effect of Extracurricular After-School Physical Activities on Academic Performance of Schoolchildren: A Cluster Randomized Clinical Trial
Source: JAMA Pediatr. 2023 Sep 18;177(11):1141–8. doi: 10.1001/jamapediatrics.2023.3615 (PMC10507588; doi:10.1001/jamapediatrics.2023.3615)
Supplement: Supplement 1. — Trial Protocol and Statistical Analysis [file jamapediatr-e233615-s001.pdf]

Structured Protocol:

Version number: 01
Date: 1 January 2017

Title of project
Effect of Extra-curricular Sports Activities After School on Primary School Children's Academic Performance

Funding: NA

Aims and objectives

Study aim: To investigate whether adding more outdoor physical activity time after school would compromise children’s academic performance in Chinese children.

Outcomes:
Primary outcome: Mathematics test scores at the end of the first academic year. The test paper will follow the design principles of International Mathematics and science learning, and referring to the local unified examination papers of grade three and grade four, the unified examination questions are formulated to evaluate the students' learning in school.

Secondary outcomes:
(1) Combined physical fitness test score at the end of the first academic year. Physical fitness test score will be determined by the total scores of physical fitness tests according to 2018 National Physical Fitness Survey Monitoring Programme. (2) Incidence of myopia in the first school year. Myopia was defined as spherical equivalent after mydriasis ≤ -0.5 D in either eye. (3) Happiness Assessment at the end of the first academic year. Happiness score is rated by Chinese version of the WHO-5 including 5 items with ratings: 0, at no time-5, all of the time. The total raw score, ranging from 0 to 25, is multiplied by 4 to give the final score, with 0 representing the worst imaginable well-being and 100 representing the best imaginable well-being. (4) Mathematics test

scores at the end of the second school year. The test paper will follow the design principles of International Mathematics and science learning, and referring to the local unified examination papers of grade three and grade four, the unified examination questions are formulated to evaluate the students' learning in school. (5) Combined physical fitness test scores at the end of the second academic year. Physical fitness test score will be determined by the total scores of physical fitness tests according to 2018 National Physical Fitness Survey Monitoring Programme. (6) Cumulative incidence of myopia over two school years. Myopia was defined as spherical equivalent after mydriasis ≤ -0.5 D in either eye. (7) Happiness assessment at the second school year. Happiness score is rated by Chinese (September, 2007) version of the WHO-5 including 5 items with ratings: 0, at no time-5, all of the time. The total raw score, ranging from 0 to 25, is multiplied by 4 to give the final score, with 0 representing the worst imaginable well-being and 100 representing the best imaginable well-being. (8) Satisfaction assessment. Satisfaction assessment.

Study hypothesis:

Primary: Children who receive 2-hour extra physical sports (the intervention group) will have comparable academic performance which is not inferior to the performance of children not receive extra physical sports (control group)

Background

With the urbanization of society, the space suitable for residents' activities is shrinking day by day, and the behavior patterns of residents have also undergone drastic changes, which is especially evident in children. The most prominent point is that the time spent outdoors by children and adolescents has been greatly shortened. At the same time, with the popularization and development of social informatization, children turn more attention to indoor sedentary activities, such as watching TV and surfing the Internet, and their dependence on video terminals is increasing. These changes have directly reduced the time for children's physical activities. In cities, children's physical activities have also shifted from unorganized and non-directional outdoor activities to organized and directional indoor activities. [1-3]

The above-mentioned changes in behaviour patterns have brought many negative effects on the physical development and physical health of children and adolescents, such as reduced exercise capacity, obesity, and myopia, which have become well-known global public health problems [1-3]. 2016 China Physical Exercise and Physical Quality - The Youth Learning Project survey found that less than 30% of students met the standard of 1 hour of moderate to vigorous physical activity (MVPA) per day. [4] According to the 1985-2010 China National Student Constitution and Health Survey, the prevalence of obesity among adolescents aged 7-19 rose from 0.2% in 1985 to 8.1% in 2010. [5] According to the survey data of the World Health Organization, the myopia rate of 15-year-old urban students in my country is as high as 78.4%. [6]

A large number of studies have confirmed that outdoor activities and physical exercise can effectively enhance the physical fitness and cardiopulmonary function of adolescents. [7,8] Physical inactivity has been shown to be a risk factor for obesity. [9-11] necessary physical exercise is not only crucial for the normal growth and development of children and adolescents, but also plays an important role in the prevention and treatment of obesity in children and adolescents and the reduction of obesity-related complications. [12] Physical exercises can effectively reduce BMI, waist circumference, body fat percentage and fat mass in obese children and adolescents, and significantly reduce systolic blood pressure. [13, 14]

Previous studies assessed the impact of outdoor activities on the development of myopia in children and adolescents. A cohort study by French et al. showed that children who spent less time outdoors had a tendency for more severe myopia. [15] A prospective randomized double-blind trial showed that adding an outdoor activity course in the school could reduce the 3-year cumulative incidence of myopia in the intervention group by 9.1% compared with the control group. [16] A multicentre, group randomized controlled trial in Taiwan found that elementary school students who spent more time outdoors (⩾200 minutes) at school had significantly less myopia progression.

Sports are closely related to the mental health of children and adolescents. Previous studies have shown that physical exercise is closely related to children's depression, anxiety, self-

esteem and cognitive function, but most of them are cross-sectional studies, and the level of evidence is low. [17, 18, 19] In general, attention to children's mental health is still quite lacking. At present, there is still a lack of strong evidence to support the correlation between performance and outdoor physical activity. Studies have shown that appropriate physical activity and outdoor sports are beneficial to improve children's classroom behavior, improve their academic performance and cognitive ability. [20, 21] However, a cluster randomized controlled trial in Norway found that an intervention to increase physical activity time among 10-year-old children did not improve academic performance.[22]

This project intends to increase the time for sports and extracurricular activities by providing students with intensive extracurricular physical education courses in schools in areas that are undergoing rapid urbanization, and follow up to observe the impact of intervention measures on children's academic performance, physical fitness, and myopia occurrence. And the influence of mental health, so as to provide a basis for the reasonable arrangement of extracurricular activities and the formulation of educational programs for students after school.

Plan of investigation

Experimental plan

Population: Pupils in the third and fourth grades of primary schools in Yudu County, Jiangxi province, China;

Sample size: The sample size was calculated based on the primary outcome and a non-inferiority margin, defined as one third of the score range of the level B (-3.3 points), which the final mean mathematic score of the control group is likely to reside. The sample size estimation was conducted based on the assumption of a 1-sided alpha level of 0.025, 90% power, an intra-class correlation of 0.10, a standard deviation (SD) of 7 for mathematics scores, and less than 5% loss to follow-up. The sample size required was 12 schools per group, or a total sample size of 24 schools. The PASS 16.0 (NCSS, LLC, US) software package was used for the calculation.

Enrolment criteria: Pupils in the third and fourth grades of primary schools in Yudu County; Voluntary participation in this study with consent and informed consent signed by guardians.

Exclusion criteria: Exclusion criteria for school: lack of a third or fourth grade; average class size less than 30 students; no conditions for two classes to play sports at the same time; Exclusion criteria for students: two or more days Monday to Friday not available to participate in studies under the project programme; unsuitability for health or physical reasons to participate in sports activities; refusal to sign informed consent.

Intervention delivery: Throughout the trial, all children will complete regular curriculums as scheduled. All curriculums will be scheduled in 2 separate semesters (from October 2020 to January 2021 and February to June 2021), with a 1-month break between these 2 semesters during which the regular curriculums and intervention will be suspended. The daily curriculums should be due by 4 p.m. on schooldays (Monday to Friday). In addition to regular curriculums, children in the intervention group will additionally receive a 2-hour (4 to 6 p.m.) extracurricular PA after school on schooldays. Children in the control group will be free to arrange their after-school time after 4 p.m.

Randomization, Interventions: Eligible participants will be randomized to one of the following intervention groups:

- *Intervention group (n=1012 children from 12 schools):* children will follow the schedule of school teaching programme. Extra-curricular sports classes will be organized and provided in this group of schools in the afternoon after school until 6 p.m. (Monday to Friday).

- *Control Model (n=1012 children from 12 schools):* children will follow the schedule of school teaching programme. Students will arrange their own after-school time after school.

- **Randomization methodology:** School-based cluster randomization will be used in this trial. All 24 schools will be stratified into 6 strata based on location (rural or urban) and baseline mathematics scores (high/medium/low). Four schools in each of the 6 strata will

be equally and randomly assigned to an intervention or control group. Randomization will be conducted by an independent data analyst using a random number generating program (www.randomization.com). Due to the nature of this intervention, masking is not feasible. However, all the investigators will be masked to the group assignments during the assignment process, and all the examiners will be masked during the examination.

Data to be acquired at baseline: Age, gender, mathematics test scores, physical fitness scores, height, weight, lung vital capacity, fifty-meter (m) sprint, sit-and-reach tests, 1-min sit-ups, and 1-min rope-jumping, spherical equivalence, axial length, behavior related questionnaire.

Follow-up schedule and follow-up data: mathematics test scores after 1 academic year and 2 academic years, physical fitness scores, height, weight, lung vital capacity, fifty-meter (m) sprint, sit-and-reach tests, 1-min sit-ups, and 1-min rope-jumping, spherical equivalence, axial length, behavior related questionnaire.

Data collection: A experienced project manager will be responsible for data collection and follow-up. The project coordinators will be thoroughly trained on the data collection forms.

Data Management Plan: All collected data will be stored and analysed in de-identified fashion by the study statistician at Zhongshan Ophthalmic Center in Guangzhou, China.

Data Analytic Plan: The primary and secondary outcomes as defined above will be compared between the intervention and control groups using intention to treat (ITT) except for the analyses regarding the incidence of myopia and relevant ocular parameters. Missing data will be imputed using the multiple imputation. For each of variables with missing value, we will use different models, selecting the predictor variables based on availability of data. We will use linear regression for continuous variables, logistic regression for binary variables, and ordinal logistic regression for ordinal variables. The multiple imputation approach will create 20 copies of the data and missing values will be imputed by chained equations. Final results will be obtained by

averaging these 20 datasets using Rubin's rules.

All analyses will take cluster effects within schools into account. The baseline distribution between the intervention and control groups in terms of continuous (age, height, weight, and sleep time), binary (gender), and ordered categorical variables (outdoor time, screen time, and study/reading time) will be compared using linear, logistic, and ordinal logistic regression models, respectively. The differences between the 2 groups in the mathematic scores, physical fitness scores, SER, and AL at baseline and at the end of the first academic year, as well as changes of these variables after 1 academic year will be assessed by linear regression models. The differences in the prevalence and incidence of myopia will be compared by proportional Z tests. Lowess plots will be performed to explore potential association between 2 continuous variables. Non-inferiority test will be used for primary outcome analysis. The statistical inference will be based on if the lower limit of the 95% confidence interval (CI) of the differences between the intervention and control groups in the mathematic scores after 1 academic year is no less than the predefined non-inferiority margin. Linear regression models will be fitted to assess factors associated with the mathematic scores after 1 academic year. Two models will be fitted with model 1 adjusting for baseline mathematic scores and cluster effect within schools and model 2 adjusting for group, cluster effect within schools, and all variables significant at the $P < 0.20$ level in model 1. A 2-sided P value < 0.05 was considered statistically significant for the remaining outcomes.

Analyzer who assessing data for the main outcome will be masked to participant's group assignment. All statistical analyses will be carried out using Stata 16 software (StataCorp, College Station TX, USA).

- Study Flow:

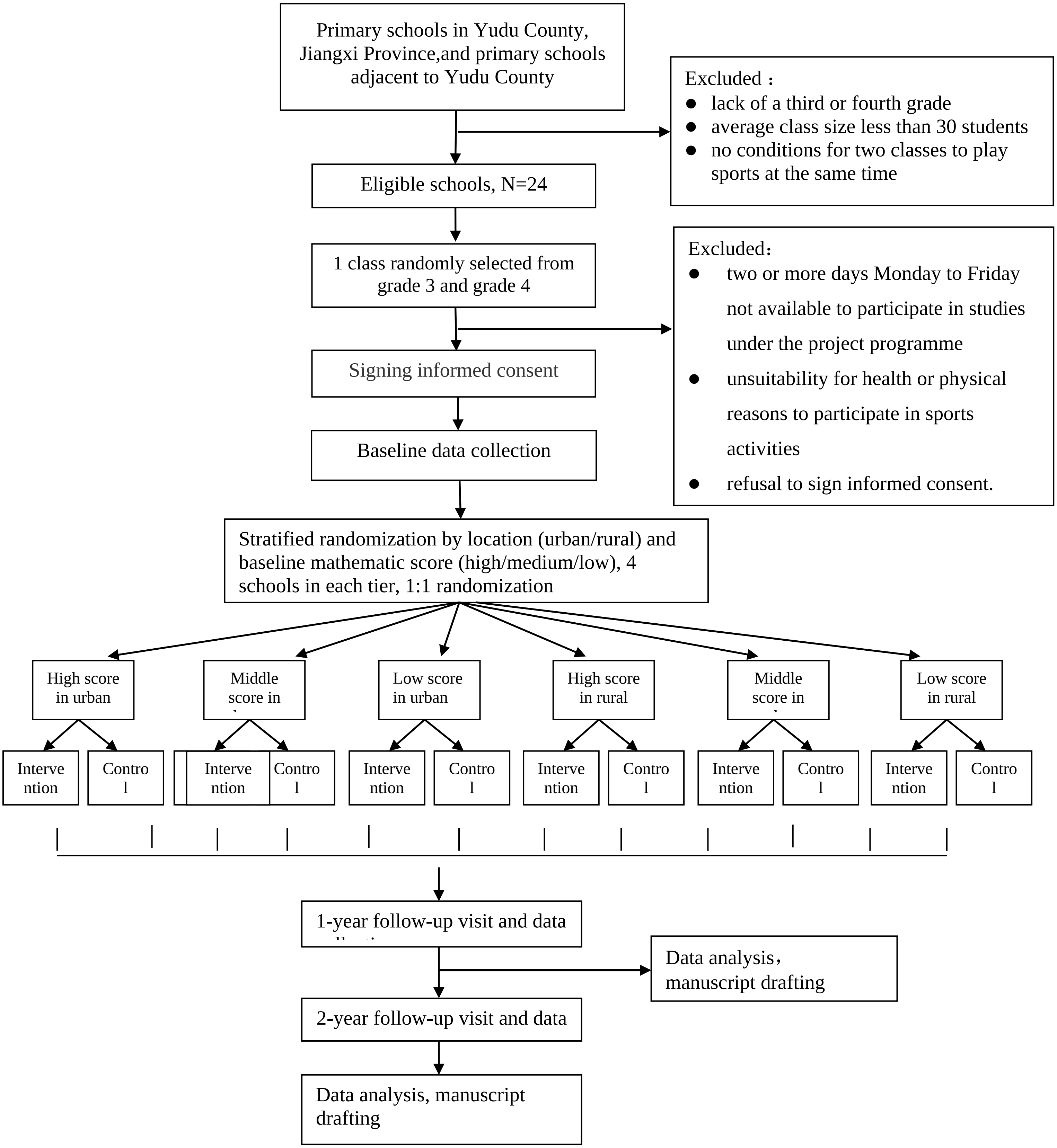
Primary schools in Yudu County, Jiangxi Province,and primary schools adjacent to Yudu County
Excluded：
lack of a third or fourth grade
average class size less than 30 students
no conditions for two classes to play sports at the same time
Eligible schools, N=24
1 class randomly selected from grade 3 and grade 4
Excluded：
two or more days Monday to Friday not available to participate in studies under the project programme
unsuitability for health or physical reasons to participate in sports activities
refusal to sign informed consent.
Signing informed consent
Baseline data collection
Stratified randomization by location (urban/rural) and baseline mathematic score (high/medium/low), 4 schools in each tier, 1:1 randomization
High score in urban
Middle score in
Low score in urban
High score in rural
Middle score in
Low score in rural
Interve ntion
Contro l
1-year follow-up visit and data
Data analysis，manuscript drafting
2-year follow-up visit and data
Data analysis, manuscript drafting

Timeline, milestones and monitoring

Study Period: December 2019 to September 2022

Number	Milestone	Time Period
1	Draft and submit project work plan and monitoring and evaluation plan (M&E plan) and preparation before start	December 2019 to September 2020
2	Recruitment and baseline data acquisition	October 2020
3	1-year follow-up visit and data acquisition	June 2021
4	Data analyzing and drafting manuscript for 1-year results	July 2021 to September 2021
5	2-year follow-up visit and data acquisition	June 2022
6	Data analyzing and drafting manuscript for 2-year results	July 2022 to September 2022

Monitoring: A dedicated Project Manager, Professor Decai Wang, will be responsible for the overall planning, implementation, coordination, and monitoring and evaluation in the project. He will coordinate all activities during the outreach screening as well as baseline and follow-up examinations at the local hospital; will ensure that appropriate planning is done for organizing and implementing screening activities; and will coordinate availability of trained manpower, equipment and facilities.

The research committee, consisted of multiple doctors and researchers from Zhongshan Ophthalmic Center (Professor Yizhi Liu, Mingguang He, Decai Wang, Dr. Yangfa Zeng), have decades of experience in cataract screening, treatment and research. All committee members have engaged in multiple prior cataract research projects in China.

Ethical considerations: Ethical approval has been received from the ethics committee of Zhongshan Ophthalmic Center (ZOC) at Sun Yat-sen University, Guangzhou, China (2020KYPJ155)

References

1. Nunes H, Silva D, Goncalves E. Prevalence and factors associated with stages of behavior change for physical activity in adolescents: a systematic review. World J Pediatr 2017;13(3):202-9.
2. Fan X, Cao ZB. Physical activity among Chinese school-aged children: National prevalence estimates from the 2016 Physical Activity and Fitness in China-The Youth Study. J Sport Health Sci 2017;6(4):388-94.
3. Australia AHK. 2016 Report Card on Physical Activity for Children and Young People., 2016.
4. Poitras VJ, Gray CE, Janssen X, et al. Systematic review of the relationships between sedentary behaviour and health indicators in the early years (0-4 years). Bmc Public Health 2017;17(Suppl 5):868.
5. Sun H, Ma Y, Han D, et al. Prevalence and trends in obesity among China's children and adolescents, 1985-2010. Plos One 2014;9(8):e105469.
6. He M, Zeng J, Liu Y, et al. Refractive error and visual impairment in urban children in southern china. Invest Ophthalmol Vis Sci 2004;45(3):793-9.
7. Gray C, Gibbons R, Larouche R, et al. What Is the Relationship between Outdoor Time and Physical Activity, Sedentary Behaviour, and Physical Fitness in Children? A Systematic Review. Int J Environ Res Public Health 2015;12(6):6455-74.
8. Grgic J, Dumuid D, Bengoechea EG, et al. Health outcomes associated with reallocations of time between sleep, sedentary behaviour, and physical activity: a systematic scoping review of isotemporal substitution studies. Int J Behav Nutr Phys Act 2018;15(1):69.
9. Bell JA, Hamer M, Batty GD, et al. Combined effect of physical activity and leisure time sitting on long-term risk of incident obesity and metabolic risk factor clustering. Diabetologia 2014;57(10):2048-56.
10. An R. Diet quality and physical activity in relation to childhood obesity. Int J Adolesc Med Health 2017;29(2).
11. Booth FW, Roberts CK, Laye MJ. Lack of exercise is a major cause of chronic diseases. Compr Physiol 2012;2(2):1143-211.
12. Jakicic JM, Davis KK. Obesity and physical activity. Psychiatr Clin North Am 2011;34(4):829-40.

13. Swift DL, McGee JE, Earnest CP, et al. The Effects of Exercise and Physical Activity on Weight Loss and Maintenance. Prog Cardiovasc Dis 2018;61(2):206-13.
14. Tan S, Chen C, Sui M, et al. Exercise Training Improved Body Composition,Cardiovascular Function, and Physical Fitness of 5-Year-Old Children With Obesity or Normal Body Mass. Pediatr Exerc Sci 2017;29(2):245-53.
15. French AN, Morgan IG, Mitchell P, Rose KA. Risk factors for incident myopia in Australian schoolchildren: the Sydney adolescent vascular and eye study. Ophthalmology 2013;120(10):2100-8.
16. He M, Xiang F, Zeng Y, et al. Effect of Time Spent Outdoors at School on the Development of Myopia Among Children in China: A Randomized Clinical Trial. JAMA 2015;314(11):1142-8.
17. Wu PC, Chen CT, Lin KK, et al. Myopia Prevention and Outdoor Light Intensity in a School-Based Cluster Randomized Trial. Ophthalmology 2018;125(8):1239-50.
18. Biddle SJ, Asare M. Physical activity and mental health in children and adolescents: a review of reviews. Br J Sports Med 2011;45(11):886-95.
19. Larun L, Nordheim LV, Ekeland E, et al. Exercise in prevention and treatment of anxiety and depression among children and young people. The Cochrane database of systematic reviews 2006(3):D4691.
20. Álvarez-Bueno C, Pesce C, Cavero-Redondo I, et al. Academic Achievement and Physical Activity: A Meta-analysis. Pediatrics 2017;140(6).
21. Trudeau F, Shephard RJ. Physical education, school physical activity, school sports and academic performance. The international journal of behavioral nutrition and physical activity 2008;5:10.
22. Resaland GK, Aadland E, Moe VF, et al. Effects of physical activity on schoolchildren's academic performance: The Active Smarter Kids (ASK) cluster-randomized controlled trial. Prev Med 2016;91:322-8.

Randomization: School-based cluster randomization will be used in this trial. All 24 schools will be stratified into 6 strata based on location (rural or urban) and baseline mathematics scores (high/medium/low). Four schools in each of the 6 strata will be equally and randomly assigned to an intervention or control group. Randomization will be conducted by an independent data analyst using a random number generating program (www.randomization.com). Due to the nature of this intervention, masking is not feasible. However, all the investigators will be masked to the group assignments during the assignment process, and all the examiners will be masked during the examination.

Data to be acquired at baseline: Age, gender, mathematics test scores, physical fitness scores, height, weight, lung vital capacity, fifty-meter (m) sprint, sit-and-reach tests, 1-min sit-ups, and 1-min rope-jumping, spherical equivalence, axial length, behavior related questionnaire.

Data Analytic Plan:

The primary and secondary outcomes as defined above will be compared between the intervention and control groups using intention to treat (ITT) except for the analyses regarding the incidence of myopia and relevant ocular parameters. Missing data will be imputed using the multiple imputation. For each of variables with missing value, we will use different models, selecting the predictor variables based on availability of data. We will use linear regression for continuous variables, logistic regression for binary variables, and ordinal logistic regression for ordinal variables. The multiple imputation approach will create 20 copies of the data and missing values will be imputed by chained equations. Final results will be obtained by averaging these 20 datasets using Rubin's rules.

All analyses will take cluster effects within schools into account. The baseline distribution between the intervention and control groups in terms of continuous (age, height, weight, and sleep time), binary (gender), and ordered categorical variables (outdoor time, screen time, and study/reading time) will be compared using linear, logistic, and ordinal logistic regression models, respectively. The differences between the 2 groups in the mathematic scores, physical fitness scores, SER, and AL at baseline and at the end of the first academic year, as well as

changes of these variables after 1 academic year will be assessed by linear regression models. The differences in the prevalence and incidence of myopia will be compared by proportional Z tests. Lowess regression plots will be performed to explore potential association between 2 continuous variables. Linear regression models will be fitted to assess factors associated with the mathematic scores after 1 academic year. Two models will be fitted with model 1 adjusting for baseline mathematic scores and cluster effect within schools and model 2 adjusting for group, cluster effect within schools, and all variables significant at the $P < 0.20$ level in model 1. A 2-sided P value < 0.05 was considered statistically significant for the remaining outcomes.

Analyzer who assessing data for the main outcome will be masked to participant's group assignment. All statistical analyses will be carried out using Stata 16 software (StataCorp, College Station TX, USA).
