# Supplementary material for: Effect of Extracurricular After-School Physical Activities on Academic Performance of Schoolchildren: A Cluster Randomized Clinical Trial
Source: JAMA Pediatr. 2023 Sep 18;177(11):1141–8. doi: 10.1001/jamapediatrics.2023.3615 (PMC10507588; doi:10.1001/jamapediatrics.2023.3615)
Supplement: Supplement 2. — eTable 1. Compliance With the Extracurricular Physical Activities in the Intervention Group eTable 2. Percentages of Extracurricular Physical Activities Implemented Outdoors, Indoors, and in Semi-Open Spaces in the Intervention Group eTable 3. Per-Protocol Analysis of Changes in Mathematic Scores and Physical Fitness After 1 Academic Year Between the Intervention and Control Groups [file jamapediatr-e233615-s002.pdf]

Supplementary Online Content

Wang D, Xiong R, Zhang J, et al. Effect of extracurricular after-school physical activities on academic performance of schoolchildren: a cluster randomized clinical trial. *JAMA Pediatr.* Published online September 18, 2023.
doi:10.1001/jamapediatrics.2023.3615

eTable 1. Compliance With the Extracurricular Physical Activities in the Intervention Group

eTable 2. Percentages of Extracurricular Physical Activities Implemented Outdoors, Indoors, and in Semi-Open Spaces in the Intervention Group

eTable 3. Per-Protocol Analysis of Changes in Mathematic Scores and Physical Fitness After 1 Academic Year Between the Intervention and Control Groups

This supplementary material has been provided by the authors to give readers additional information about their work.

© 2023 Wang D et al. *JAMA Pediatr.*

eTable 1. Compliance with the extracurricular physical activities in the intervention group.

Grade	Term	Month for intervention	Number of scheduled sessions	Number of successful sessions	Number of unsuccessful sessions due to weather	Number of unsuccessful sessions due to school events	Compliance, %
3	2020 last term	Oct/Nov/Dec/Jan	1111	1072	32	7	96.5
3	2021 first term	Feb/Mar/Apr/May/June	1256	1207	40	9	96.1
4	2020 last term	Oct/Nov/Dec/Jan	1111	1072	32	7	96.5
4	2021 first term	Feb/Mar/Apr/May/June	1246	1197	40	9	96.1
		**Total**	4724	4548	144	32	96.3

© 2023 Wang D et al. *JAMA Pediatr.*

eTable 2. Percentages of extracurricular physical activities implemented outdoors, indoors, and in semi-open spaces in the intervention group.

Grade	Term	Month for intervention	Number of successful sessions	Number of outdoor sessions	Number of sessions implemented in semi-open spaces	Number of indoor sessions	Rate of outdoor sessions, %
3	2020 last term	Oct/Nov/Dec/Jan	1072	859	103	110	80.1
3	2021 first term	Feb/Mar/Apr/May/June	1207	960	156	91	79.5
4	2020 last term	Oct/Nov/Dec/Jan	1072	858	103	111	80.0
4	2021 first term	Feb/Mar/Apr/May/June	1197	949	156	92	79.3
		**Total**	4548	3626	518	404	79.7

© 2023 Wang D et al. *JAMA Pediatr.*

eTable 3. Per-protocol analysis of changes in mathematic scores and physical fitness after 1 academic year between the intervention and control groups.

	Study group, N (%)		Mean Difference （95%CI）[a]	*P* Value
	Intervention	Control		
**Mathematics scores**				
All participants	952 (50.0)	951(50.0)	1903 (100)	
Baseline	74.64 (16.90)	74.94 (16.43)	-0.30 (-1.76, 1.17)	
1 academic year	78.78 (17.75)	77.70 (16.33)	1.07 (-0.37, 2.52)	0.48
1 academic year - baseline	4.14 (13.21)	2.77 (14.87)	1.37 (0.13, 2.61)	
**Overall Physical fitness scores**				
All participants	952 (50.0)	951(50.0)	1903 (100)	
Baseline	68.69 (9.02)	68.89 (9.02)	-0.20 (-1.07, 0.66)	
1 academic year	78.15 (7.12)	75.51 (7.79)	2.64 (1.93, 3.35)	0.002[c]
1 academic year - baseline	9.52 (6.07)	6.72 (7.45)	2.80 (2.14, 3.47)	
**Prevalence of myopia**				
All participants [b]	730 (48.9)	764 (51.1)	1494 (100)	
Baseline	89 (12.2)	95 (12.4)	-0.24 (-8.99, 8.94)	
1 academic year	137 (18.8)	156 (20.4)	-1.65 (-12.34, 9.56)	
**Incidence of myopia**	48/641 (7.5)	61/669 (9.1)	-1.63 (-9.69, 6.26)	>0.99[c]

Data are mean (standard deviation).

[a] Linear regression models assessed the differences between the 2 groups in the mathematics scores and physical fitness scores at baseline and at the end of 1 academic year, as well as changes in these variables after 1 academic year. All analyses took into account cluster effects within schools.

[b] Children with ocular abnormalities, unsuccessful cycloplegia at baseline and follow-up visit, and lost to follow-up were excluded from the analysis. The differences in the prevalence and incidence of myopia were compared by proportional Z tests adjusting for cluster effect.

[c] Bonferroni correction was applied to multiple comparisons for secondary outcomes (physical fitness scores and myopia incidence), and the corrected *P* value was presented.

© 2023 Wang D et al. *JAMA Pediatr.*
