# Supplementary material for: Effect of Extracurricular After-School Physical Activities on Academic Performance of Schoolchildren: A Cluster Randomized Clinical Trial
Source: JAMA Pediatr. 2023 Sep 18;177(11):1141–8. doi: 10.1001/jamapediatrics.2023.3615 (PMC10507588; doi:10.1001/jamapediatrics.2023.3615)
Supplement: Supplement 3. — Data Sharing Statement [file jamapediatr-e233615-s003.pdf]

Data Sharing Statement

Wang. Effect of Extracurricular After-School Physical Activities on Academic Performance of Schoolchildren. *JAMA Pediatr*. Published September 18, 2023.
doi:10.1001/jamapediatrics.2023.3615

Data
Data available: Yes
Data types: Deidentified participant data
How to access data: All data will be available from the corresponding authors, contacted at zengyangfa@qq.com
When available: With publication

Supporting Documents
Document types: None

Additional Information
Who can access the data: researchers whose proposed use of the data has been approved
Types of analyses: For a specified purpose
Mechanisms of data availability: with a signed data access agreement
